# Spontaneous Refractory Intercostal Artery Hemorrhage in a Patient With Decompensated Liver Cirrhosis

**DOI:** 10.7759/cureus.87325

**Published:** 2025-07-05

**Authors:** Jiro Kimura, Prakash Chauhan, Calvin Eriksen, Matthew Cooper, Raj Prasad

**Affiliations:** 1 Division of Transplant Surgery, Medical College of Wisconsin, Milwaukee, USA

**Keywords:** liver cirrhosis, liver failure, liver transplantation, spontaneous intercostal artery hemorrhage, transcatheter arterial embolization

## Abstract

Intercostal artery hemorrhage (ICAH) is an uncommon occurrence that may lead to significant clinical complications if not promptly addressed. We present the case of a 39-year-old man with a history of liver cirrhosis who was waitlisted for liver transplantation (LT). He presented with acute, severe left shoulder pain without any recent trauma. Contrast-enhanced computed tomography revealed a left posterolateral chest wall intramuscular hematoma with active contrast extravasation originating from the left sixth intercostal artery. Interventional radiology angiography confirmed the extravasation, and embolization was successfully performed to achieve hemostasis. Despite the embolization, the patient required blood transfusions due to a gradually decreasing hemoglobin level. Five days after the initial procedure, CT angiography identified additional multifocal small arterial hemorrhages. Subsequently, the patient developed acute-on-chronic liver failure. Fortunately, a deceased donor LT was performed eight days after admission. Postoperatively, his coagulation status normalized, and no further bleeding was observed. His recovery was uneventful, with no recurrence of ICAH, and he was discharged on postoperative day 9. This case highlights an acutely decompensated cirrhotic patient who developed spontaneous ICAH due to coagulopathy, successfully treated with LT. Given the potential severity of ICAH in high-risk patients, prompt and intensive initial management is essential.

## Introduction

Intercostal artery hemorrhage (ICAH) is a rare but potentially life-threatening condition [1]. Severe coughing is recognized as a major contributing factor in some cases [2]. The most common underlying cause of ICAH is trauma, which may include blunt or penetrating injuries, as well as iatrogenic causes such as thoracentesis or surgical procedures [3]. While spontaneous rupture of the intercostal artery can occur, it is much less frequent and typically associated with underlying conditions such as neurofibromatosis type 1, coarctation of the aorta, connective tissue disorders (e.g., Marfan syndrome), or severe hypertension [4]. The overall incidence of ICAH is extremely low and cannot be reliably quantified from current literature, with most data derived from isolated case reports and small series.

According to the literature, undiagnosed or untreated intercostal artery injury can result in massive hemothoraces. While initial tamponade may occur, rebleeding is possible, potentially leading to recurrent or retained hemothorax and hemodynamic instability [5]. Fatal outcomes have been reported, particularly when bleeding is not promptly recognized and managed, as ICAH can be both life-threatening and technically challenging to control, even with surgical intervention.

To the best of our knowledge, ICAH leading to hepatic decompensation, defined by the new onset of ascites, hepatic encephalopathy, variceal bleeding, or jaundice, in a cirrhotic patient has not been previously reported [6]. Herein, we present a case of a cirrhotic patient who fully recovered from refractory ICAH following liver transplantation (LT).

## Case presentation

A 39-year-old man with a history of liver cirrhosis secondary to cryptogenic cirrhosis presented to the emergency department with complaints of acute, severe left shoulder pain without any recent injury. He was triaged as level 4 according to the Emergency Severity Index [7]. His past medical history included hypertension, hepatic encephalopathy, ascites, esophageal varices, and portal vein thrombosis. His Model for End-Stage Liver Disease 3.0 score was 24 points, and he had been on the waiting list for LT for six months [8]. He was not taking any antithrombotic medications.

**Figure 1:**
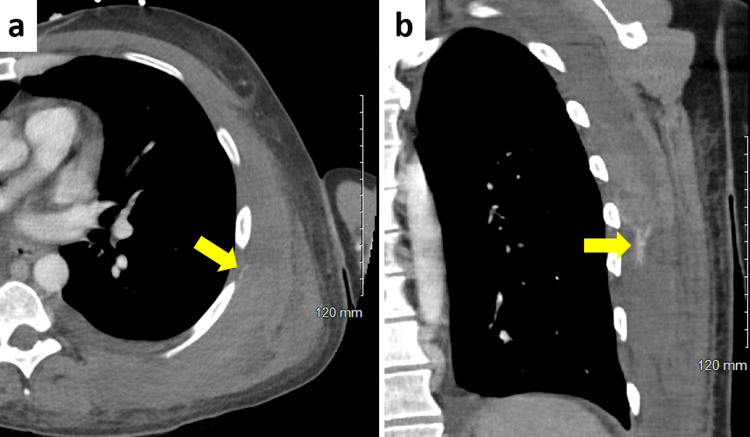
Contrast-enhanced abdominal CT scan showing contrast extravasation at the level of the sixth intercostal space (arrow): (a) axial view and (b) coronal view

He denied having a fever, cough, or chest pain. On examination, his vital signs were within normal limits. He appeared jaundiced and had a bruise with tenderness on his left back. Laboratory results showed a drop in hemoglobin from 8.6 g/dL three days earlier to 6.4 g/dL and a prothrombin time international normalized ratio of 2.1. A shoulder X-ray revealed no acute fracture or dislocation, and the regional soft tissues appeared intact. Contrast-enhanced CT revealed an intramuscular hematoma in the left posterolateral chest wall with active contrast extravasation originating from the left sixth intercostal artery (Figure 1).

**Figure 2:**
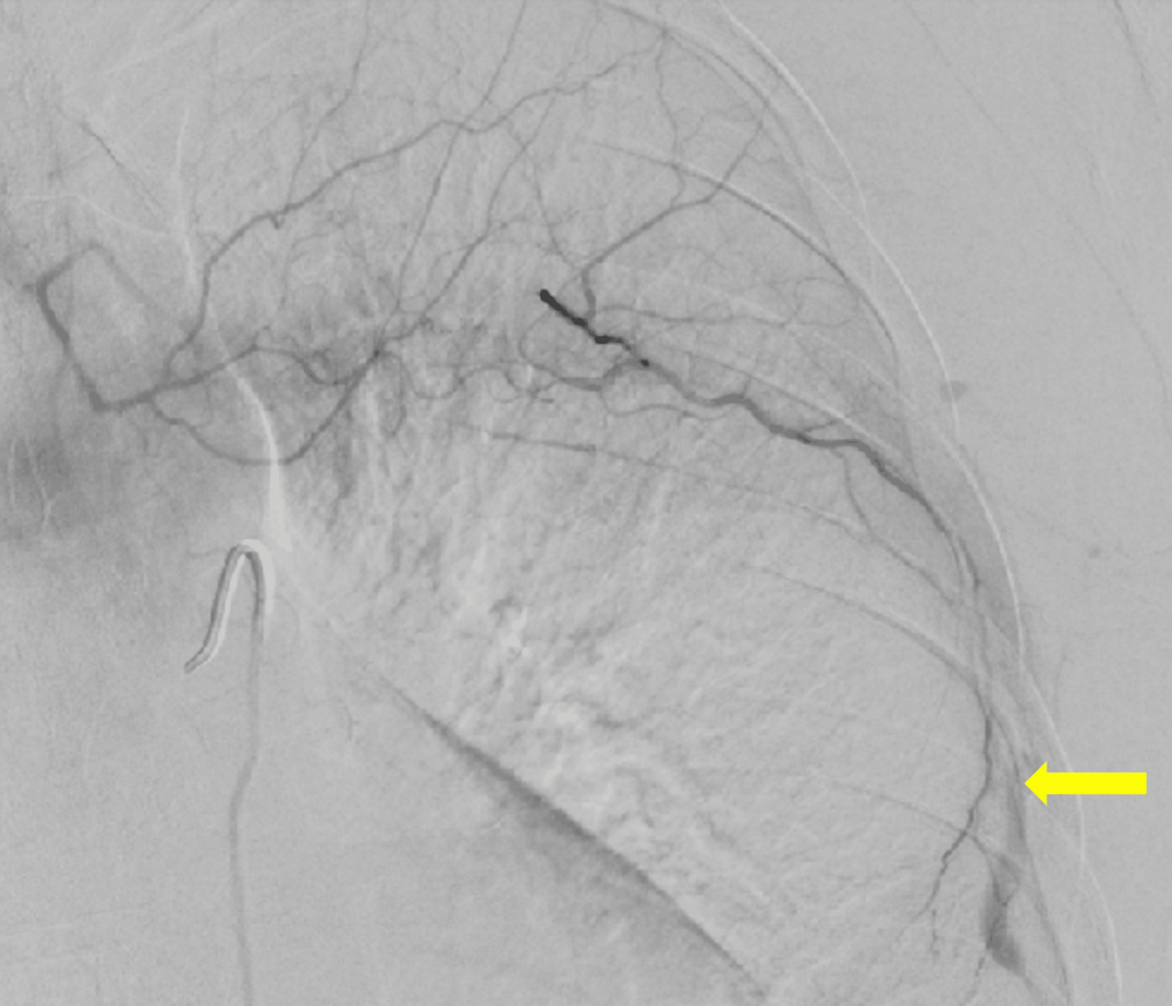
Interventional radiology and TAE The ICAH was identified, and TAE was subsequently performed. ICAH, intercostal artery hemorrhage; TAE, transcatheter arterial embolization

He was admitted to our hospital, and interventional radiology was consulted for arterial embolization. Angiography revealed contrast extravasation from the left sixth intercostal artery (Figure 2). Embolization was performed, and hemostasis was achieved. However, he continued to require blood transfusions due to a gradual decline in hemoglobin levels. Five days after the initial embolization, CT angiography was performed and revealed additional multifocal small arterial hemorrhages. A compression binder was applied to his chest wall, which provided partial effectiveness.

Despite these interventions, he developed acute-on-chronic liver failure. His bilirubin and creatinine levels progressively increased from 6.3 mg/dL and 0.73 mg/dL to 15.8 mg/dL and 1.49 mg/dL, respectively (Table 1). Fortunately, a deceased donor LT was performed eight days after admission. Following the surgery, his coagulation status normalized, and no postoperative hemorrhage occurred. His postoperative course was uneventful, with no recurrence of ICAH, and he was discharged on postoperative day 9. 

**Table 1:** Blood test results at hospital admission and at the time of LT LT, liver transplantation; PT-INR, prothrombin time international normalized ratio

Test	Reference range	At hospital admission	At the time of LT
White blood cells (10³/μL)	3.9-11.2	8.9	5.4
Hemoglobin (g/dL)	13.7-17.5	6.4	7.8
Hematocrit (%)	40-51	19	23
Platelets (10³/μL)	165-366	67	47
Sodium (mmol/L)	136-145	138	141
Potassium (mmol/L)	3.4-5.1	3.6	2.9
Bilirubin (mg/dL)	0.2-1.2	6.3	15.8
Creatinine (mg/dL)	0.70-1.30	0.73	1.49
PT-INR	0.8-1.1	2.1	2.1

## Discussion

Table 2 summarizes the reported cases of spontaneous ICAH [1,2,5,9-17]. Since the first case was described in 2005, only 13 cases, including the present one, have been reported to date. The median patient age was 58 years (IQR: 45-69), with a male predominance (10 males and two females). The most common presenting symptom was pain (12 out of 13 patients). Identified contributing factors included cough (n = 3), cirrhosis (n = 1), hypertension (n = 1), systemic lupus erythematosus (n = 1), and hemodialysis (n = 1). Interestingly, four patients had no identifiable contributing factors. Three patients presented in shock following the hemorrhage.

**Table 2:** Summary of reported cases with spontaneous ICAH ICAH, intercostal arterial hemorrhage; SLE, systemic lupus erythematosus; TAE, transcatheter arterial embolization

No.	Author	Year	Age	Sex	Symptoms	Hemodynamic status	Bleeding site	Diagnostic imaging	Ruptured intercostal artery	Laterality	Treatment	Complications	Prognosis	Contributing factor	Antithrombotic medications
1	Dua et al. [1]	2014	47	Male	Right chest pain and dyspnea	Shock	Right thorax	CT	11th	Right	Thoracotomy	None	Alive	None	None
2	Sangani and Naliath [2]	2020	60	Male	Left chest and abdominal pain	Stable	Left chest wall and thorax	CT	8th	Left	Surgery	None	Alive	Cough	None
3	Jang et al. [5]	2015	39	Male	Left flank pain	Stable	Left retroperitoneum	CT	11th	Left	Conservative	None	Alive	Cough	None
4	Yu et al. [9]	2006	76	Male	Suddenly enlarging mass	Stable	Right abdominal wall	CT	10th	Right	TAE	None	Alive	Cough	None
5	Moon et al. [10]	2008	45	Male	Right flank pain	Stable	Retroperitoneum	CT	10th and 11th	Right	TAE	None	Alive	Hypertension	None
6	Mathew et al. [11]	2008	69	Male	Left chest pain	Shock	Left thorax and abdominal wall	CT	10th	Left	TAE	None	Alive	Unknown	None
7	Lu et al. [12]	2012	50	Female	Left shoulder pain	Stable	Left shoulder	CT	4th	Left	TAE	Alveolar hemorrhage	Died of alveolar hemorrhage	SLE	Enoxaparin
8	Dobrilovic et al. [13]	2013	62	Female	Chest and back pain	Shock	Left thorax	MRI	NA	Left	TAE	NA	Alive	Unknown	None
9	Ishida et al. [14]	2014	58	Male	Back pain	Stable	Posterior mediastinum	CT	10th	Right	Conservative	None	Alive	None	None
10	Junck and Utarnachitt [15]	2015	63	Male	Back pain	Stable	Posterior mediastinum	CT	8th	Right	TAE	None	Alive	None	None
11	Afonso et al. [16]	2020	76	NA	Right shoulder pain	Stable	Right subscapular region	CT	5th	Right	TAE	None	Alive	Hemodialysis	Acetylsalicylic acid
12	Izumoto [17]	2022	48	Male	Back pain	Stable	Posterior mediastinum	CT	9th	Right	TAE	None	Alive	None	None
13	Our case	2025	39	Male	Left shoulder pain	Stable	Left chest wall	CT	6th	Left	TAE	Liver failure	Alive	Cirrhosis	None

The most common cause of ICAH is trauma [18]. Other reported etiologies include rupture of intercostal artery aneurysms associated with conditions such as neurofibromatosis type 1 and coarctation of the aorta, as well as anticoagulant use, bleeding disorders, pulmonary infections, and uncontrolled hypertension [4]. Some authors have also documented cases of spontaneous ICAH occurring without a clear inciting event, such as trauma, or in the absence of identifiable arterial wall pathology or associated systemic disease [1]. Notably, to the best of our knowledge, this is the first reported case of ICAH leading to acute-on-chronic liver failure in a patient with cirrhosis.

Except for one patient who underwent magnetic resonance imaging due to a contrast allergy, all other cases were diagnosed using CT. Hemorrhage occurred on the left side in six patients and on the right side in seven. The majority of patients were treated with transcatheter arterial embolization (TAE) (n = 9). Eight patients recovered without complications. However, our patient developed liver failure due to recurrent ICAH following TAE. One patient, who had systemic lupus erythematosus and was receiving enoxaparin, died due to alveolar hemorrhage.

Fortunately, the patient underwent LT eight days after the onset of ICAH. Following LT, his coagulation status normalized, and no further bleeding was observed. However, in patients with a high risk of bleeding, ICAH may be fatal due to hemorrhage that is refractory to standard treatment, although the overall mortality rate of this condition remains unknown [5]. In such cases, rapid and intensive management, including correction of coagulopathy, is essential.

## Conclusions

We presented the case of a cirrhotic patient who developed ICAH as a result of abnormal coagulation, ultimately progressing to liver decompensation. Despite initial management with TAE, the patient experienced recurrent hemorrhage and acute-on-chronic liver failure, illustrating the challenging and refractory nature of ICAH in patients with cirrhosis. Fortunately, urgent LT successfully corrected the underlying coagulopathy and resolved the bleeding, leading to full recovery. This case highlights the importance of prompt and aggressive intervention, including correction of coagulopathy, in cirrhotic patients with ICAH, as the condition may be life-threatening in this vulnerable population.
